# Dried Blood Spots for Measuring *Vibrio cholerae*-specific Immune Responses

**DOI:** 10.1371/journal.pntd.0006196

**Published:** 2018-01-29

**Authors:** Anita S. Iyer, Andrew S. Azman, Malika Bouhenia, Lul O. Deng, Cole P. Anderson, Michael Graves, Pavol Kováč, Peng Xu, Edward T. Ryan, Jason B. Harris, David A. Sack, Francisco J. Luquero, Daniel T. Leung

**Affiliations:** 1 Division of Infectious Diseases, Department of Internal Medicine, University of Utah School of Medicine, Salt Lake City, UT, United States of America; 2 Department of Epidemiology, John Hopkins University, Baltimore, MD, United States of America; 3 Médecins Sans Frontières, Geneva, Switzerland; 4 Department of Pandemic and Epidemic Diseases, World Health Organization, Juba, South Sudan; 5 National Public Health Laboratory, Republic of South Sudan Ministry of Health, Juba, South Sudan; 6 National Institute of Diabetes, Digestive and Kidney Diseases (NIDDK), Laboratory of Bioorganic Chemistry (LBC), National Institutes of Health, Bethesda, Maryland, United States of America; 7 Division of Infectious Diseases, Department of Medicine, Massachusetts General Hospital, Boston, MA, United States of America; 8 Department of Medicine, Harvard Medical School, Boston, MA, United States of America; 9 Department of Immunology & Infectious Diseases, Harvard T.H. Chan School of Public Health, Boston, MA, United States of America; 10 Department of Pediatrics, Harvard Medical School, Boston, MA, United States of America; 11 Department of International Health, John Hopkins University, Baltimore, MD, United States; 12 Epicentre, Paris, France; 13 Division of Microbiology & Immunology, Department of Pathology, University of Utah School of Medicine, Salt Lake City, UT, United States of America; The Johns Hopkins University, UNITED STATES

## Abstract

**Background:**

*Vibrio cholerae* causes over 2 million cases of cholera and 90,000 deaths each year. Serosurveillance can be a useful tool for estimating the intensity of cholera transmission and prioritizing populations for cholera control interventions. Current methods involving venous blood draws and downstream specimen storage and transport methods pose logistical challenges in most settings where cholera strikes. To overcome these challenges, we developed methods for determining cholera-specific immune responses from dried blood spots (DBS).

**Methodology/principal findings:**

As conventional vibriocidal assay methods were unsuitable for DBS eluates from filter paper, we adopted a drop-plate culture method. We show that DBS collected from volunteers in South Sudan, and stored for prolonged periods in field conditions, retained functional vibriocidal antibodies, the titers of which correlated with paired serum titers determined by conventional spectrophotometric methods (r = 0.94, p = 0.00012). We also showed that eluates from DBS Serum Separator cards could be used with conventional spectrophotometric vibriocidal methods, and that they correlated with paired serum at a wide range of titers (r = 0.96, p<0.0001). Similarly, we used ELISA methods to show that *V*. *cholerae* O-specific polysaccharide antibody responses from DBS eluates correlated with results from paired serum for IgG (r = 0.85, p = 0.00006), IgM (r = 0.79, p = 0.00049) and IgA (r = 0.73, p = 0.0019), highlighting its potential for use in determination of isotype-specific responses. Storage of DBS cards at a range of temperatures did not change antibody responses.

**Conclusion:**

In conclusion, we have developed and demonstrated a proof-of-concept for assays utilizing DBS for assessing cholera-specific immune responses.

## Author summary

Cholera remains a major public health issue among underprivileged populations in the developing world. Current methods of disease surveillance are inadequate for identifying key populations at highest risk of cholera. Serosurveillance can provide accurate measurements of an individual or population’s exposure to cholera infection or oral cholera vaccine (OCV) induced immunity, though they require venous blood draw and stringent processing needs. Dried blood spots (DBS) overcome these challenges, acting as a portable surveillance tool suitable for field use. We developed a drop-plate culture method for evaluating vibriocidal and cholera-specific isotype responses using DBS from OCV-immunized volunteers from South Sudan. Blood equivalent to only two drops were spotted on Whatman Protein Saver (WPS) DBS cards. Vibriocidal titers from WPS eluates determined by drop-plate culture methods correlated well with serum based assays. In addition, by using DBS cards capable of automatic separation of serum from blood, we demonstrate that vibriocidal titers and *V*. *cholerae* polysaccharide antibody responses could be measured by conventional spectrophotometric methods and that these responses are stable over a range of storage temperatures. In summary, we show that cholera-specific immune responses can be measured using DBS, providing a potential tool for large-scale serosurveillance field studies for cholera.

## Introduction

Despite efforts to improve access to clean water and sanitation in resource-poor settings, cholera remains a significant public health threat globally. Identifying key populations at the highest risk of cholera is essential to prioritize public health interventions, including vaccination and efforts to improve water and sanitation. Furthermore, understanding the impact of cholera control programs requires monitoring of evidence of cholera transmission. Current surveillance methods, largely based on health facility reports of acute watery diarrhea, with infrequent laboratory confirmation of the pathogen, are inadequate. Detection and quantification of immune responses in serum (serosurveillance) can provide a new avenue for rapid and accurate assessments of recent cholera exposure; and such an approach could play a central role in estimating risk and transmission in a population [1]. However, serosurveys are limited by the need for venipuncture, refrigeration, and the processing of biohazardous samples, which may be difficult to achieve in the resource-poor settings where cholera occurs.

Assays of antibody responses adapted to use dried blood spots (DBS) overcome the above challenges by 1) alleviating the need for venipuncture, 2) reducing the costs associated with blood collection, supplies, labor, transportation and storage, 3) being relatively stable at ambient temperatures for extended periods, and 4) reducing the amount of blood required. Although the first use of DBS dates back to 19^th^ century, contemporary widespread use of DBS was pioneered by Robert Guthrie as a diagnostic tool for neonatal screening of phenylketonuria [2, 3]. More recently, DBS have been used for screening of metabolic disorders, detection of pathogens, and drug testing, in various sample types including blood, saliva, breast milk and stool [4–6]. Several studies have demonstrated the utility of DBS for serological and molecular detection of a number of infectious diseases, including tetanus [7], diphtheria [8], HIV [9–11], hepatitis A, B, C and E [12–15], dengue [16, 17], polio [18] and measles [19]. The performance characteristics of DBS tests have approached that of serum assays, and their low costs and convenience have resulted in widespread use and endorsement by the WHO [20]. Despite the extensive use of DBS in a wide range of settings, including its use for microbial detection of *Vibrio cholerae* in stool [21], the use of DBS for *V*. *cholerae*-specific antibody responses has yet to be determined. Here we demonstrate the development and provide proof-of-concept of methods for the determination of cholera-specific immune responses, including vibriocidal titers, and *V*. *cholerae* polysaccharide-specific isotype antibodies using DBS.

## Results

### Functional antibodies to V. cholerae are retained in dried blood spots and their vibriocidal titers correlate to those obtained by venipuncture

**Fig 1:**
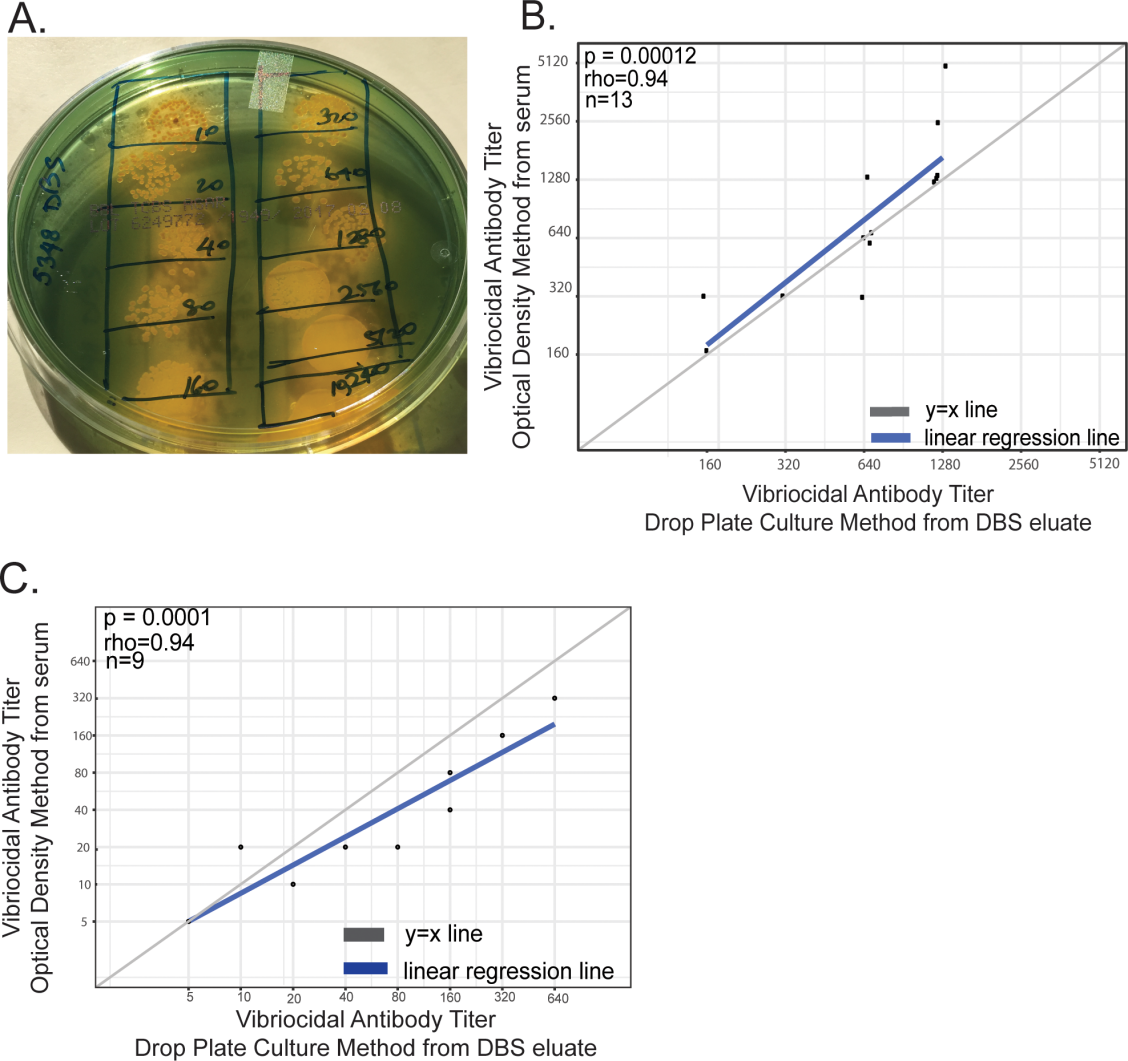
Determination of vibriocidal titers from dried blood spots collected using Whatman 903 protein (WPS) saver cards and drop-plate culture method. A. Representative image of vibriocidal titer obtained by drop-plate method using eluate from DBS WPS card obtained from a recipient of an oral cholera vaccine in South Sudan (with a known serum titer of 1280). B. Spearman correlation of vibriocidal titers determined using eluates from DBS WPS cards containing blood collected from recipients of an oral cholera vaccine in South Sudan, by drop-plate culture method with titers obtained from paired serum using conventional spectrophotometric method. C. Spearman correlation of vibriocidal titers determined using eluates from DBS WPS cards containing blood spiked with mAbA2 IgG, by drop-plate culture method with titers obtained from paired serum using conventional spectrophotometric method.

The vibriocidal antibody is the most commonly used marker of protection against cholera and exposure to *V cholerae*. We examined whether dried blood spots (DBS) (panel A in S1 Fig) retained such functional antibodies after extended storage at ambient temperatures in Sub-Saharan Africa. We collected DBS from recipients of an oral cholera vaccine in South Sudan, and stored and shipped the cards at ambient temperatures. We found that the conventional method for determining vibriocidal titers using spectrophotometry could not be performed due to color perturbations from the presence of heme in eluates from Whatman 903 protein saver cards (WPS cards, panel B in S1 Fig). We thus decided to test alternate methodologies for endpoint detection and optimized a drop-plate (or spot-titer) culture method [22], defining vibriocidal titers as reciprocal of highest dilution of DBS eluates that resulted in reduction in growth (either clumped colonies or serrated margins of growth) as compared to an eluate-free control (confluent bacterial growth). To ensure we were working with samples that were positive for vibriocidal titers, we opted for pre- and post- vaccination DBS samples from volunteers whose vibriocidal titers from matched serum were moderate to high (>160), as determined previously [23]. Using this method, we could see inhibition in bacterial growth (fewer colonies) at higher eluate concentrations (lower dilutions) and confluent growth at lower eluate concentrations (higher dilutions) (Fig 1A). We found that vibriocidal titers of DBS WPS eluates from vaccinees as determined by drop-plate culture correlated with paired serum samples determined by spectrophotometry (Fig 1B, rho = 0.94, p = 0.00012, n = 13). These results demonstrate that functional vibriocidal antibodies from DBS cards that underwent prolonged periods of storage in field conditions in South Sudan are detectable, and that the titers from these DBS cards matched serum titers determined by conventional spectrophotometric method.

To confirm that drop-plate culture works for a range of vibriocidal antibody concentrations, we used blood samples from healthy volunteers with no evidence of historical exposure to *V cholerae* (negative for vibriocidal titers), spiked with varying concentrations of a human monoclonal antibody (mAb) against *V*. *cholerae* O-specific polysaccharide (OSP), IgG mAb [24]. We used this monoclonal antibody because vibriocidal responses predominantly target the *V*. *cholerae* OSP [25, 26]. We found that vibriocidal titers determined by culture method from spiked mAb DBS WPS eluates correlated strongly with paired serum titers determined by spectrophotometry (Fig 1C, rho = 0.94, p = 0.0001, n = 9).

### DBS serum separator cards are a convenient alternative for the determination of vibriocidal titers by conventional methods

**Fig 2:**
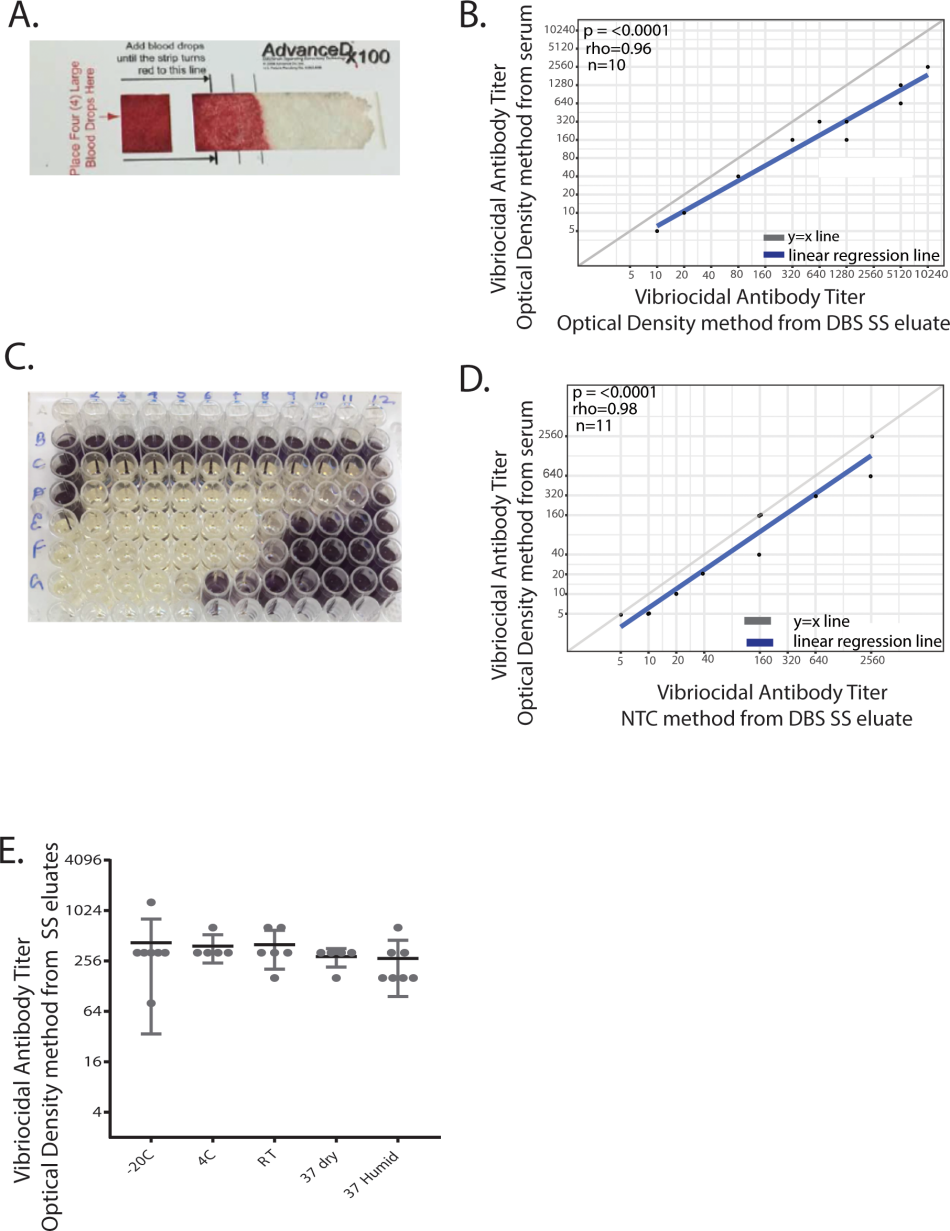
Use of dried blood spot serum separator cards for the determination of vibriocidal titers using conventional methods. A. Representative image of serum automatically separated from dried blood spots on a commercial (AdvanceDx 100) serum separator card. B. Spearman correlation of vibriocidal titers determined using eluates from DBS SS cards spotted with blood containing mAbA2 IgG with titers obtained from paired serum using conventional spectrophotometric method. C. Representative image of vibriocidal titer obtained by chromogenic dye NTC method using eluates from DBS SS cards. Wells B1,C1,D1 represent eluate-free control; wells E1,F1,G1 represent saline controls; row B2-12 represents two-fold dilution of unspiked sample controls; wells C2, D2, E2, F2, G2 represents eluates of blood spiked with mAbA2 IgG at 12,500ng/ml, 6250ng/ml, 3125ng/ml, 1526ng/ml and 1000ng/ml respectively, while rows C, D, E, F, G 3–12 represents two-fold dilutions of the respective concentrations from column 2. D. Spearman correlation of vibriocidal titers determined using eluates from DBS SS cards spotted with blood containing mAbA2 IgG with titers obtained from paired serum using chromogenic dye reduction method. E. Vibriocidal titers from spiked DBS SS card eluates stored for one week at a range of temperatures.

Recent technological developments have enabled the rapid and automatic separation of serum from blood on bound glass filters. We investigated whether DBS Serum Separator (DBS SS) cards could be used for determination of vibriocidal titers by conventional spectrophotometric methods. We used AdvanceDx 100 card (Advance Dx, Inc, Fig 2A) and spotted them with blood spiked with increasing concentrations of the OSP monoclonal antibody mAb IgG (blood was drawn from volunteers with no known history of cholera). We found that vibriocidal titers from eluates of DBS collected on the SS cards correlated with those from paired plasma samples (Fig 2B, r = 0.96, p ≤0.0001, n = 10), demonstrating the utility of DBS SS cards as an alternate tool for determination of vibriocidal titers by conventional spectrophotometric methods.

Vibriocidal assays using the chromogenic dye neotetrazolium chloride (NTC) serves as a visual colorimetric assay for the determination of titers without a need for spectrophotometer [27]. In this assay, viable bacteria reduce colorless NTC to a purple colored compound upon growth and titers can be determined based on growth (purple color) or lack of growth (no color change) (Fig 2C). We found that titers of DBS SS card eluates determined by NTC method corresponded to plasma titers determined by conventional spectrophotometric method (Fig 2D, r = 0.98, p ≤0.0001, n = 11).

To evaluate DBS SS suitability for use in field settings, we tested the stability of vibriocidal antibodies on DBS SS cards over a range of temperatures. We stored the DBS SS cards at 4°C, -20°C, room temperature (~22°C), and 37°C (both non-humidified and humidified conditions) for one week and examined the effect on vibriocidal titers. To create the humidified condition, a pan with water was placed in a 37°C incubator, while the non-humidified condition was devoid of the same. No significant differences in vibriocidal titers were found after a week of storage at these different temperatures (Fig 2E). These results demonstrate that DBS SS cards could be used to determine vibriocidal titers from DBS samples using both spectrophotometric and chromogenic dye-based methods, and that they are stable across a range of storage temperatures and humidity conditions.

### Determination of Vibrio cholerae O-specific polysaccharide (OSP) responses using DBS eluates

**Fig 3:**
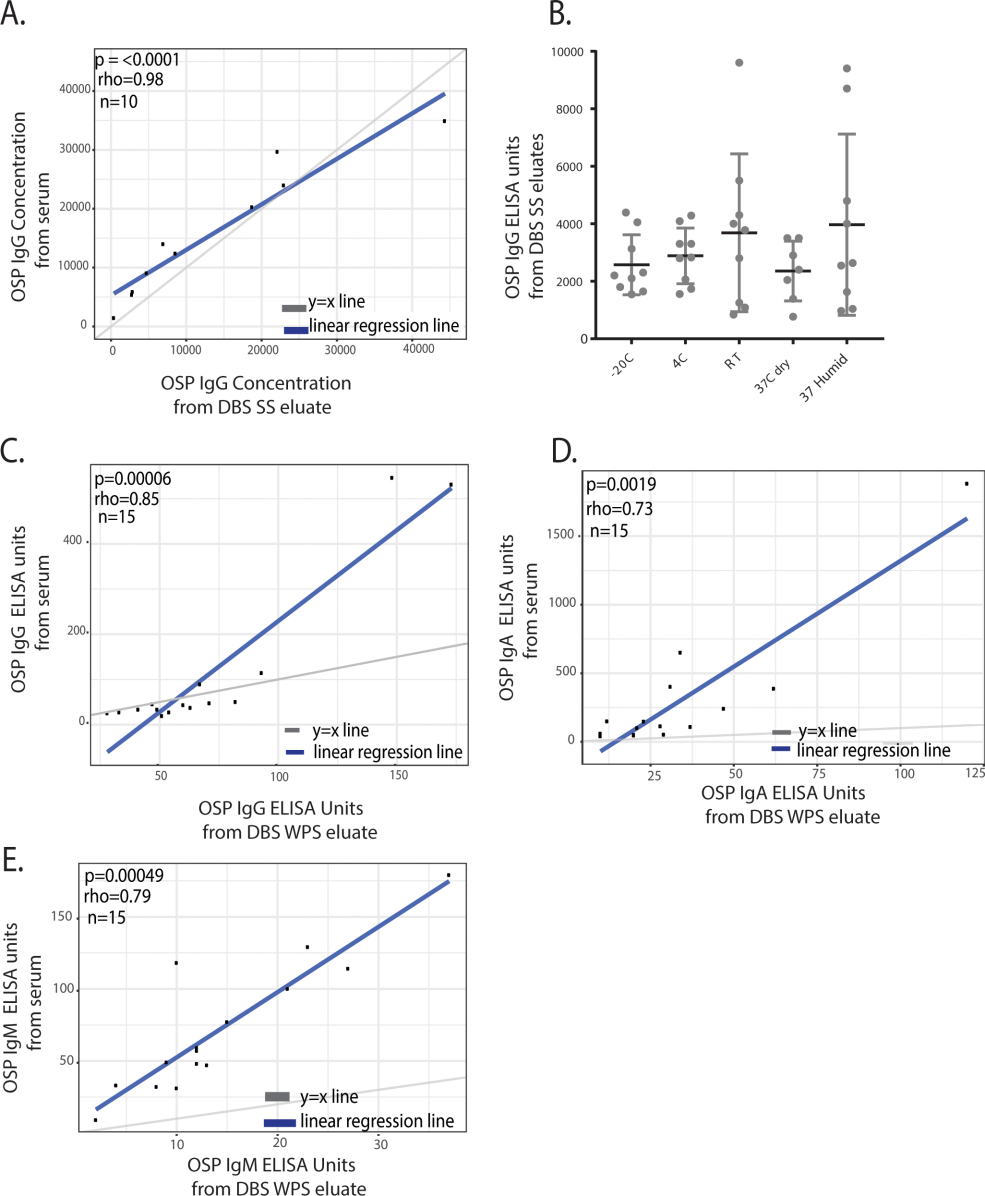
Use of DBS WPS and DBS SS cards for the determination of V. cholerae OSP-specific antibody responses. A. Spearman correlation of *V*. *cholerae* OSP-specific IgG responses between serum and paired DBS SS card eluates, obtained from blood spiked with OSP monoclonal antibody at different concentrations B. OSP-specific IgG responses from spiked DBS SS card eluates stored for one week at a range of temperatures. C-E. Spearman correlations of *V*. *cholerae* O1 Inaba OSP-specific IgG (B), IgA (C), and IgM (D) antibody responses between serum and paired DBS WPS card eluates, obtained from oral cholera vaccine recipients in South Sudan at day 21 after vaccination.

Immunity to *Vibrio cholerae* is serogroup-specific, with serogroup defined by the O-specific polysaccharide (OSP) component of lipopolysaccharide (LPS) [25]. Polysaccharide-specific responses have been shown to be associated with protection against cholera in studies of household contacts of cholera patients [28, 29]. Thus, we investigated the use of DBS eluates for determination of OSP-specific isotype antibody responses in both laboratory- and field-derived samples. We first spiked whole blood from volunteers without known *V*. *cholerae* exposure with the OSP-specific monoclonal antibody at a wide range of dilutions and compared OSP-specific antibody responses, as determined by ELISA, between serum samples and those obtained from paired DBS SS eluates (Fig 3A, r = 0.98, p <0.0001). We found significant correlations between the two methods. As with vibriocidal assays, we did not find any significant differences in OSP-specific IgG responses after a week of storage of DBS SS cards at different experimental temperatures (Fig 3B, n = at least 7 in each group).

As a pilot application of this method in field settings, we used samples from 15 South Sudanese vaccinees for whom we had paired DBS WPS and serum samples. We saw significant correlations between OSP titers from DBS WPS and serum, as determined by ELISA (Fig 3C–3E; IgG: r = 0.85, p = 0.00006, IgA r = 0.73, p = 0.0019, IgM r = 0.79, p = 0.00049).

## Discussion

Existing methods of cholera surveillance rely on clinical reporting of cases of acute watery diarrhea, with occasional confirmation by microbial detection. Serosurveillance is recognized as an important public health and scientific tool to aid in estimation of pathogen exposure and disease risk [30], although practical use of serosurveillance for cholera using conventional methods is limited by venous blood draw and stringent downstream storage and transport requirements. DBS eliminates expertise required for venipuncture, and is potentially both cost effective and time sensitive, important factors while considering outbreak response measures. In this study, we developed several methods for the determination of functional and quantitative antibody responses to cholera using DBS. We provide proof-of-concept using DBS samples collected from cholera vaccine recipients in an internally displaced persons camp in South Sudan as well as DBS from spiked blood experiments. These methods could be used for detecting cholera infection as well as estimating oral cholera vaccine (OCV) responses.

We found that conventional vibriocidal antibody methods could not be performed on DBS WPS eluates due to interference of heme on spectrophotometric readings. We thus adapted the vibriocidal assay to a drop-plate culture technique, a method modified for use in limited resource settings. Using a gridded plate containing a highly selective media for *Vibrio spp*., serial dilutions of the vibriocidal assay can be tested on a single plate compared to the conventional spread plate culture technique where each dilution requires a separate plate [31]. The use of the drop-plate culture method has a number of additional advantages over conventional methods: it does not require a spectrophotometer, does not require colony counting [32], and is compatible with DBS WPS eluates. We showed that DBS samples from South Sudan, stored for 1–2 months at ambient temperatures in the field, followed by 9 months at -20°C, retained functional bactericidal antibodies, quantifiable in the form of vibriocidal titers. We also show that DBS WPS titers were within a two-fold dilution of matched sera for the selected subset of positive samples (titers 160–1280). Two-fold differences in titers are common in the conventional serum vibriocidal assay methods and within the acceptable range of variability. Notably, while the correlation was statistically significant (r = 0.94; p = 0.00012), it was not perfect. Furthermore, we did not examine samples with titers higher than the aforementioned range, such as those which would be achieved through infection, and thus DBS-based assays outside such a range needs further evaluation.

Despite its advantages, DBS drop-plate culture is more labor-intensive compared to spectrophotometric methods. Thus, we investigated other potential field-ready solutions that could enable the high-throughput assessment of cholera-specific immune responses. We tested the use of commercial DBS SS cards that provide on-card automatic separation of serum from DBS. We optimized and validated this approach using blood spiked with human monoclonal antibodies against the O-specific polysaccharide of *V*. *cholerae*. Vibriocidal and OSP-specific antibody titers from SS eluates correlated with those from serum. Further, by using NTC dye-based endpoints, we show that the vibriocidal assay using DBS SS cards could be used in conditions where a specialized agar may not be available. Overall, these data suggest that DBS SS cards can be a convenient alternative to DBS WPS cards for cholera-specific antibody measurements.

While vibriocidal responses are the best characterized serological marker of protection against cholera [33], the complexity of such a bactericidal assay makes it less desirable for low-resource settings. Thus, we explored the use of ELISA methods on DBS eluates to measure antibody responses. Vibriocidal responses are predominantly targeted against the *V*. *cholerae* O-specific polysaccharide (OSP), a component of LPS that determines serogroup specificity [25]. Post vaccination OSP responses correlate with vibriocidal titers [25, 26], and studies of household contacts have also shown that antibodies against both OSP are associated with protection [29]. We found, using eluates from both WPS and SS cards that OSP-specific responses determined from DBS correlate with those determined from paired serum. Thus, ELISA methods can be used on DBS to determine OSP-specific responses, offering a simplified alternative to complex vibriocidal assays for determination of cholera-specific immune responses.

We examined the field-readiness of storage of DBS cards for cholera serosurveillance. We showed that DBS WPS samples from South Sudan, stored in field conditions in Africa for months, transported by standard commercial shipping company at ambient temperatures before storage in the laboratory, retained functional bactericidal antibodies, quantified as vibriocidal antibody titers. We also showed that storage of WPS and SS cards at a range of temperatures did not impact polysaccharide-specific or vibriocidal antibody responses, suggesting it may be favorable for use in field settings.

Our study has several limitations. In this proof-of-concept study, we selected DBS samples from volunteers immunized with oral cholera vaccine who had serum vibriocidal titers up to 2560 [23], responses that are expected for OCV, but lower than that usually achieved following natural infection. Validation with samples with a serum vibriocidal titer outside this range is needed, though our experiments with blood spiked with monoclonal antibody shows an ability to detect titers linearly across a large range of titers. Secondly, we validated our vibriocidal findings with the *V*. *cholerae* strain Ogawa X25049 as a representative serotype. Our methods may require optimization at the bacterial input level while working with other serotypes. While our results with DBS were comparable to those of serum-based approaches for the range of titers tested in laboratory settings, additional field testing is warranted in hyper-endemic and outbreak prone regions, where there may be more dramatic temperature and humidity conditions than those tested here. Also, while serum and South Sudan WPS eluates showed statistically significant correlations for OSP-specific antibody responses, they were not perfect. It is possible that OSP-specific isotype antibody responses for WPS eluates were at the lower limits of detection. Further application in field settings with samples from naturally infected persons with titers higher than those seen in OCV recipients, as well as those with asymptomatic or mildly symptomatic infection whose titers may be lower than that tested in our study, is warranted.

In conclusion, we have developed a microtiter assay using DBS (requiring only two drops, approximately 100μL, of blood) for the detection of vibriocidal and *V*. *cholerae* OSP-specific isotype antibodies. Potential uses include cholera serosurveillance and assessment of OCV immunogenicity in resource-limited settings.

## Materials and methods

### DBS collection, transport, and storage from persons receiving the oral cholera vaccine in South Sudan

We collected serum samples pre- (day 0) and post- (day 21 and 35) vaccination as a part of an OCV immunogenicity study in an internally displaced persons camp in Juba, South Sudan [23]. Blood (collected either via venipuncture or by finger prick from volunteers) was spotted on Whatman 903 protein saver cards (Whatman 903, GE Healthcare, LifeSciences). We collected a total of 5 blood spots per DBS card from each volunteer for each time point (S1 Fig). Samples reached approximately 50% saturation per spot. Each DBS card was put in a biohazard plastic bag with dessicator and stored at ambient temperature in sub-Saharan Africa. The median storage days for the samples used were 70, 47 and 34 days, for pre-vaccination (D0) and post-vaccination days 21 and 35, respectively. Samples were then shipped by postal service to the University of Utah where they were stored at -20°C for approximately 9 months or longer until analysis.

### Elution of antibodies from DBS Whatman 903 Protein Saver (DBS WPS) cards

We punched out a total of 4 saturated fragments using a 6mm diameter hole puncher (Office Depot Brand Single-Hole Punch) from a total of 4 dried blood-soaked spots. We added 4 fragments from the same volunteer to a microfuge tube and eluted overnight with 200 μl PBS + 0.05% Tween 20 elution buffer on a shaker set at 100rpm. Our pilot experiments showed detectable titers regardless of elution of 1 or 4 spots (upon using 50μl elution buffer per punched fragment). Elution of 2 to 4 spots yielded titers that were only 2-fold different (acceptable range of variation in the field) from each other while elution of 1 spot yielded 4-fold lower titers. To ensure that we had sufficient eluates for the different antibody assays and that the elution time was consistent, we proceeded with eluting 4 spots per sample. Eluates were spun at 15,000 rpm for 5 min, supernatants transferred to new tubes and subsequently used for assays.

### Quantification of vibriocidal antibodies in DBS WPS eluates using drop-plate culture techniques

We assigned undiluted DBS WPS eluates a starting titer of 10. We performed two-fold serial dilutions in a 96 well, flat bottom plate. We grew target strain *V*. *cholerae* O1 Ogawa (X25049) on blood agar. Colonies were inoculated the next day in 10 ml Brain Heart Infusion (BHI) broth and grown to a mid-log phase O.D. of 0.3 (~3 hours at 37°C, 220rpm) as per procedures of conventional vibriocidal assay. For DBS eluates, a 1:10 dilution of this inoculum proved optimal for the detection of neutralizing antibodies in a concentration linear manner. Serum, but not DBS, samples were heat inactivated at 56°C for 30 mins to eliminate human complement-induced titer variations between volunteers. An external source of guinea pig complement (Sigma Aldrich, catalog # S1639, final dilution 1:10) was added to the assay plates. A mixture of bacteria, guinea pig complement, and saline were then added to the 96 well plate containing serially diluted DBS eluates and incubated at 37°C for 1 hour. 10μl of this mix per well were spotted into hand-drawn, labelled grids (drop-plate culture method) in a plate with thiosulfate citrate bile sucrose (TCBS) agar. The vibriocidal titer by drop-culture method was defined as the highest dilution that yields fewer clumped colonies or a serrated margin of growth, as compared to confluent growth. To compare with DBS eluate titers, we also repeated vibriocidal assays using paired serum samples by conventional methods. For conventional vibriocidal assays involving serum and spectrophotometric methods [34], bacteria were first adjusted at an O.D. of 0.3, then used at a 1:5 final dilution in assay. Titers were defined as the reciprocal of serum dilution that resulted in ≥50% reduction in O.D. compared to serum free controls.

### Elution of serum from DBS WPS and serum separator (SS) cards spotted with blood containing V. cholerae O-specific polysaccharide (OSP) monoclonal antibody

To establish a method of determining vibriocidal titers from DBS through a traditional spectrophotometric method, we used a commercial DBS SS card, the AdvanceDx 100 (provided in kind from Advance Dx, Inc). Blood was collected in acid citrate dextrose anti-coagulant tubes and spiked with a monoclonal antibody against *V*. *cholerae* OSP IgG at a starting concentration of 1000 ng/ml. Spiked blood was two-fold serially diluted. Unspiked blood served as the negative control. 200 μl of blood were spotted on individual cards and left at room temperature for a period of 3–4 hours. Cards were subsequently put in dessicator bags and stored at RT for at least 24 hours prior to use. The on-card-separated sera were then punched out using a 6mm diameter puncher. On average, we could punch out 4 saturated fragments per SS card. Similarly, for WPS cards, 50μl of spiked blood was spotted per spot for a total of 4 spots per card. Spots were eluted in PBS+0.05% Tween 20 buffer (50μl buffer/punched spot) overnight at RT on orbital shaker set at 100rpm. Blood not used for DBS spotting was spun at 2000rpm for 10mins for plasma.

### Quantification of vibriocidal antibodies from DBS SS card eluates

We conducted vibriocidal assays using DBS SS cards spotted with blood spiked with OSP monoclonal antibody as described above and with paired serum samples as per conventional methods [34]. DBS SS card eluates were assigned an arbitrary starting titer of 10 while serum was heat inactivated and diluted 1:10 for use. For determination of titers using the chromogenic dye based method, we used a mix of neotetrazolium chloride: sodium succinate solution and defined titers as the highest serum dilution that did not show a color change to purple based on methodology described previously [27].

### V. cholerae O-specific polysaccharide (OSP)-specific antibody ELISA

For OSP ELISAs, we selected DBS cards from 15 South Sudan OCV recipients paired for the study timepoints day 0, 21 and 35[23]. Four saturated fragments/ volunteer/ timepoint were punched and added onto a labeled 24 well plate. Samples were eluted overnight using 500μl phosphate buffer saline with 0.05% Tween (elution buffer) on a room temperature shaker set at 100rpm. Eluates were used for ELISAs the next day. DBS eluates and positive control (pooled sera) were diluted as required and added to *V*. *cholerae* Ogawa or Inaba OSP: bovine serum albumin conjugate coated plates after blocking. *V*. *cholerae* OSP:BSA was prepared as previously described [35]. Goat anti-human horseradish peroxidase (HRP) IgG, IgM and IgA conjugates were used for detection. Plates were developed with O-phenylenediamine dihydrochloride (OPD) substrate in the presence of H_2_O_2_, read kinetically at 450nm for 5 min and milli-absorbance units/min were obtained as described previously [26]. OSP readings of DBS eluates were normalized with an internal control (pooled sera sample) incorporated in every plate to account for inter-experimental variation.

### Statistical analyses

Spearman’s correlation test was used for assessing correlations. We used the Kruskal-Wallis test for evaluating vibriocidal and OSP-specific responses across temperatures. Two-tailed p-values of ≤ 0.05 were considered statistically significant.

### Ethics statement

The study protocol was reviewed and approved by the ethical review committee of the South Sudan Ministry of Health and the institutional review boards of Johns Hopkins Bloomberg School for Public Health and the University of Utah and carried out in accordance with relevant guidelines and regulations. Written informed consent was obtained from all study participants. In cases of participants between the age of 8 and 17 years, an assent was obtained in addition to the written, signed parental consent form.

## Supporting information

S1 Fig Whatman 903 protein saver cards and elution. A. Representative image of a DBS sample on a WPS card obtained from a volunteer immunized during an oral cholera vaccine campaign in Sudan.
B. Representative image of eluates from DBS WPS cards from cholera vaccinees serially diluted across a 96-well plate, depicting the red color perturbations from heme.
(PDF)

S1 Text Vibriocidal asssay protocol. Detailed experimental protocol of vibriocidal assay using SS cards, WPS cards and serum.
(DOCX)

S1 Data RawData. Raw data of all the experiments included in the manuscript.
(XLSX)
