# Supplementary figures and images for: Dried Blood Spots for Measuring Vibrio cholerae-specific Immune Responses
Source: PLoS Negl Trop Dis. 2018 Jan 29;12(1):e0006196. doi: 10.1371/journal.pntd.0006196 (PMC5805362; doi:10.1371/journal.pntd.0006196)

A.

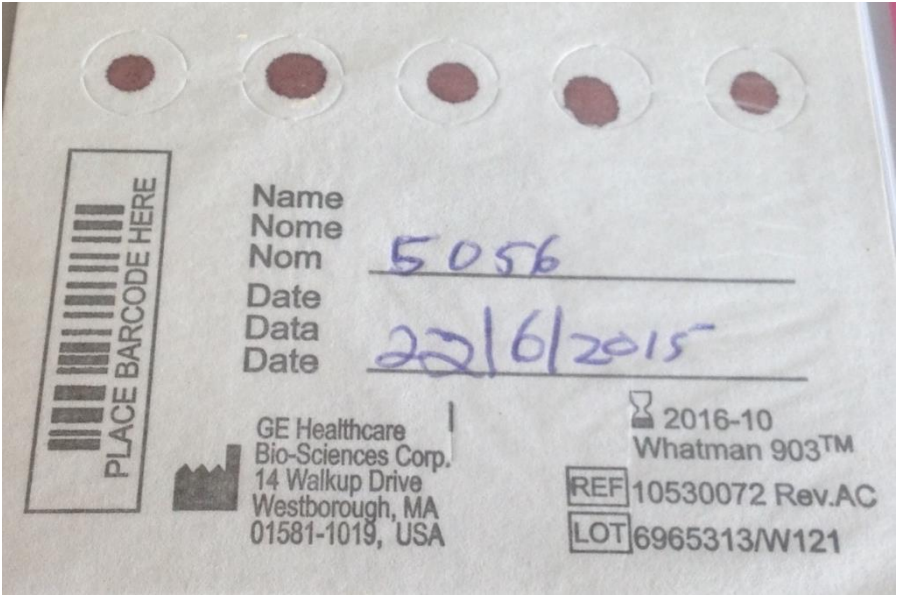

B.

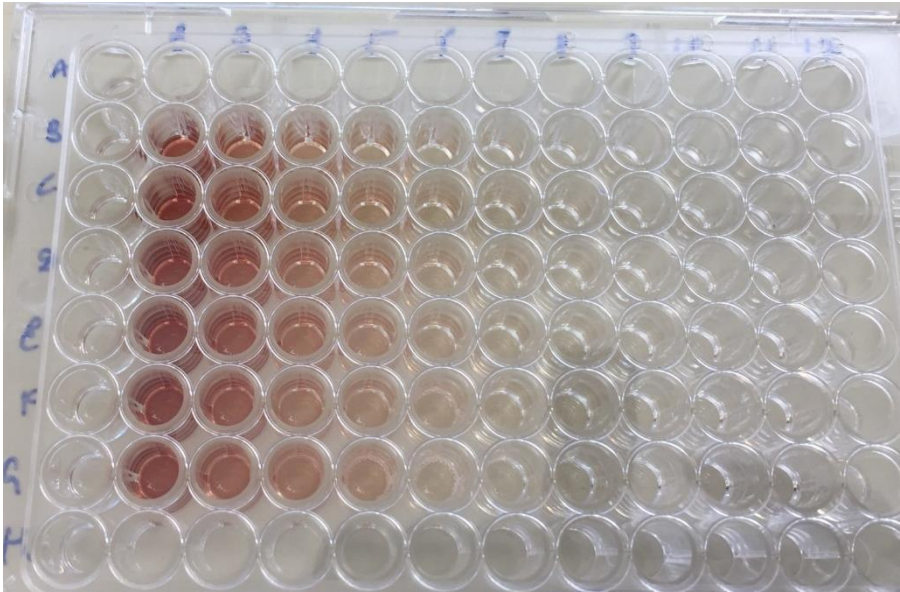

Supplement: S1 Fig — A. Representative image of a DBS sample on a WPS card obtained from a volunteer immunized during an oral cholera vaccine campaign in Sudan. B. Representative image of eluates from DBS WPS cards from cholera vaccinees serially diluted across a 96-well plate, depicting the red color perturbations from heme. (PDF) [file pntd.0006196.s001.pdf]
