# Supplementary material for: Dried Blood Spots for Measuring Vibrio cholerae-specific Immune Responses
Source: PLoS Negl Trop Dis. 2018 Jan 29;12(1):e0006196. doi: 10.1371/journal.pntd.0006196 (PMC5805362; doi:10.1371/journal.pntd.0006196)
Supplement: S1 Text — Detailed experimental protocol of vibriocidal assay using SS cards, WPS cards and serum. (DOCX) [file pntd.0006196.s002.docx]

**Aim:** Determine vibriocidal titers by serum O.D., DBS-WPS culture and Adx100-O.D. method in parallel.

This helps avoiding any differences in results that may arise due to different bacteria or complement aliquots.

**Protocol:**

**I]** **Night before you do vibriocidal assay, perform steps A, B, C and D.**

**A]** **Culture:** Streak *Vibrio cholerae* O1 (X25049 El Tor Ogawa) on blood agar plates. Incubate at 37°C overnight.

**B]** **Serum Preparation:** Aliquot 20 µl serum in microfuge tubes and heat inactivate at 56°C for 30 mins in water bath or heat block. Save in 4C for next day use.

**C]** **DBS WPS elution:** Punch 4 spots using 6mm diameter punch/ DBS sample. Put it an eppendorf tube and push it to the corner using forceps. Add 200µl elution buffer (PBS+ 0.05% Tween 20). Elute overnight on shaker at 100rpm. Centrifuge next day at max rpm for 5 mins. Transfer supernatant to new labelled tubes. Use this eluate directly for your assay.

Note: We do not heat inactivate eluates due to precipitation issues. We assume the eluate to have an arbitrary dilution of 1:10.

**D] Adx100 SS card elution):** Cut the separated serum portion out using scissors. Punch 4 spots with 6mm diameter punch / SS card. Put it an eppendorf tube and push it to the corner using forceps. Add 200µl elution buffer (PBS+ 0.05% Tween 20). Elute overnight on shaker at 100rpm. Centrifuge next day at max rpm for 5 mins. Transfer supernatant to new labelled tubes. Use this eluate directly for your assay.

Note: We do not heat inactivate eluates due to precipitation issues. We assume the eluate to have an arbitrary dilution of 1:10.

**II] On the day of assay:**

1. Inoculate a loopful of bacteria from the blood agar plates in a 15ml culture tube with 10ml sterile BHI medium. Incubate on shaker set at 220rpm, 37°C for 3 hours.
2. Centrifuge the culture at 3000rpm for 10 minutes. Decant supernatant and resuspend the pellet in 10ml sterile normal saline and pipette up and down several times.
3. Repeat wash one more time. Resuspend in a final volume of 1ml saline. Perform a bunch of dilutions in eppendorf tubes each time you do assay.

1:10 dilution: 900µl saline + 100 µl *Vibrio cholera*

1:15 dilution: 933µl saline + 67 µl *Vibrio cholera*

1:20 dilution: 950µl saline + 50 µl *Vibrio cholera*

1:25 dilution: 960µl saline + 40 µl *Vibrio cholera*

1. **Checking bacterial O.D:** In a 96 well plate, add 200 µl saline in a well as a negative control. Add 200 µl of the above dilution to respectively labeled wells. Read at 600nm. Check which dilution corresponds to 0.3 O.D. at 600nm using spectrophotometer. Plus or minus 0.02 is fine. If it’s beyond that, do additional dilutions and pick the one that corresponds to 0.3. O.D.

For serum and Adx100-WPS O.D. methods: Use the bacterial dilution that corresponds to 0.3 O.D. as the inoculum

For WPS-DBS culture method: Use 1:10 dilution of 0.3 O.D. as the inoculum for growth controls.

1. Dispense 25µl of cold saline in all columns of a 96 well plate except column 2.
2. Add 50µl of 1:10 diluted serum or undiluted Adx100/WPS samples in column 2 based on individual desired plate layouts and dilute it to 1:2 across.

(That is, Add 25 µl from column 2 to column 3. Mix 25 µl from column 3 to 4 until column 12. Discard extra 25 µl from column 12).

At this point, all wells should have 25 µl.

Example Plate layout for let’s say sample ‘A’ by serum and Adx100 method

Plate 1

|  | 1 | 2 | 3 | 4 | 5 | 6 | 7 | 8 | 9 | 10 | 11 | 12 |
| --- | --- | --- | --- | --- | --- | --- | --- | --- | --- | --- | --- | --- |
| A | GC |  |  |  |  |  |  |  |  |  |  |  |
| B | GC | Serum A 1:10 |  |  |  |  |  |  |  |  |  |  |
| C | GC | Serum A 1:10 |  |  |  |  |  |  |  |  |  |  |
| D | GC | Adx100 A |  |  |  |  |  |  |  |  |  |  |
| E | Saline | Adx100 A |  |  |  |  |  |  |  |  |  |  |
| F | Saline |  |  |  |  |  |  |  |  |  |  |  |
| G | Saline |  |  |  |  |  |  |  |  |  |  |  |
| H | Saline |  |  |  |  |  |  |  |  |  |  |  |

Plate 2:

|  | 1 | 2 | 3 | 4 | 5 | 6 | 7 | 8 | 9 | 10 | 11 | 12 |
| --- | --- | --- | --- | --- | --- | --- | --- | --- | --- | --- | --- | --- |
| A | GC |  |  |  |  |  |  |  |  |  |  |  |
| B | GC | WPS A |  |  |  |  |  |  |  |  |  |  |
| C | GC | WPS A |  |  |  |  |  |  |  |  |  |  |
| D | GC |  |  |  |  |  |  |  |  |  |  |  |
| E | Saline |  |  |  |  |  |  |  |  |  |  |  |
| F | Saline |  |  |  |  |  |  |  |  |  |  |  |
| G | Saline |  |  |  |  |  |  |  |  |  |  |  |
| H | Saline |  |  |  |  |  |  |  |  |  |  |  |

1. Prepare indicator. Thaw complements on ice two hours before you get to this step. Never re-use thawed complement aliquots.
2. Remember to use the right inoculum for O.D. versus culture method as explained before while preparing indicator. Prepare growth controls (indicators). Per 96 well plate calculation will be as follows.

| Indicator | Saline | Diluted Bacteria | Other |
| --- | --- | --- | --- |
| Growth control | 2.55ml | 150 µl | 300 µl Guinea pig complement |
| Saline | 2.65ml | N/A | 300 µl Guinea pig complement |

Use immediately after preparation.

1. Add 25 µl of growth control (indicator) in column 2-12. Add 25 µl to A1, B1, C1 and D1. Add 25 µl saline E, F1, G1 and H1.

All wells should now have 50 µl.

1. Incubate at 37°C for 1 hour with shaker on at 50rpm.
2. Add 150 µl sterile BHI broth to the serum, adx100 containing plates. Incubate at 37°C for 2 hours, no shake.
3. For the WPS containing plates, after 1 hour, spot 10 µl from each well into TCBS plates with labelled grids and let dry for 10 mins. Incubate at 37°C overnight. Read results next day.

10

20

40

80

160

320

640

1280

2560

5120

10,240

GC

|  | | |
| --- | --- | --- |
|  |  |  |
|  | |  |

1. For serum, Adx100 SS card eluate plates, after the 2 hours BHI incubation, read at 595nm. Average the O.D values of four GC wells. Divide the value by half. For the sample wells, determine which wells correspond to or is less than the 50% GC value.
2. For WPS plates: Compare reduction in growth compared to GC spot.

**Materials and Reagents:**

**Culture tested:** Ogawa X25049

**Complement used:** Sigma Aldrich, cat no 51639-5ml, Lot #SLBN4787V

**Others**

1. T5ml, T15, T50ml sterile tubes

2. 1000µl, 200µl autoclaved tips

3. Multichannel 100 and 200µl pipettes

4. Individual pipettes: 10, 20, 200 and 1000µl

5. Platinum wire loop

6. 70% alcohol

7. Shaker

8. Water bath/ heat block set at 56°C. Make sure there is a thermometer in it.

9. Autoclaved eppendorf tubes.

10. Flat bottom Microplate: Nunc Delta Surface, cat no # 167008 and Lot no # 137647

12. Dedicated 6mm puncher

13. Kimwipes

14. Dedicated Scissors

15. PBS + Tween 0.05%

16. Shaker at 100rpm.

17. st. BHI broth

18. sterile TCBS plates, st. 5% sheep blood agar plates

19. sharpies

20. sterile saline 0.9%

21. centrifuge that can spin at 3000rpm

22.microcentrifuge

23. guinea pig complement
